# Air quality at venues of mixed smoking policies in Kazakhstan

**DOI:** 10.18332/tid/127230

**Published:** 2020-09-15

**Authors:** Jamilya Sadykova, Ardak Baizhaxynova, Byron Crape

**Affiliations:** 1 National Coalition ‘Smoke-free Kazakhstan’, Nur-Sultan, Kazakhstan; 2 Public Health Program, School of Medicine, Nazarbayev University, Nur-Sultan, Kazakhstan

**Keywords:** designated smoking areas, smoking policy, air quality, Kazakhstan

## Abstract

**INTRODUCTION:**

Enclosed designated smoking areas (DSAs) and smoking zones are allowed in food-serving venues in Kazakhstan. Air quality in smoke-free food-serving venues, in venues with smoking throughout, in those with DSAs and those with smoking zones, is not fully understood.

**METHODS:**

A cross-sectional study with aim to evaluate PM_2.5_ concentrations in the venues with mixed smoking was conducted from September to October 2017 in Almaty, the largest city of Kazakhstan. A total of 44 rooms within the selected 29 venues were evaluated: 100% smoke-free (5), non-smoking zones (7), smoking zones (7), non-smoking venues with DSAs (8), venues that allow smoking throughout (9), and DSAs (8). Real-time PM_2.5_ measurement was conducted by TSI SidePak AM510 Personal Aerosol Monitor and used to rank health-risk assessment using the Air Quality Index developed by the U.S. Environmental Protection Agency.

**RESULTS:**

Smoke-free food-serving venues had moderate levels of air quality with mean PM_2.5_ of 26.2 μg/m^3^ while non-smoking zones inside venues that also permitted smoking had a mean of 56.5 μg/m^3^, corresponding to unhealthy air quality. Venues restricting smoking only to DSAs also had unhealthy air quality in non-smoking areas (mean PM_2.5_ = 87.6 μg/m^3^) while DSAs had hazardous levels of air quality (mean PM_2.5_ = 647.9 μg/m^3^). Smoking zones inside the venues and venues allowing smoking throughout had a mean PM_2.5_ of 180.3 and 182.0 μg/m^3^, respectively, ranking as very unhealthy. On average 3.5 persons were observed in DSAs with mean volume of 38.9 m^3^. Cigarette and/or hookah were the major source of PM_2.5_. The higher the smoker density the poorer the air quality in the venue.

**CONCLUSIONS:**

Hazardous level of PM_2.5_ due to tobacco products inside DSAs demonstrated the low efficiency of a smoking ban with exemptions. A complete smoking ban in food-serving venues should be in place to fully protect people from hazardous air quality conditions.

## INTRODUCTION

Secondhand smoke (SHS) is a combination of sidestream (smoke emitted from the tobacco stick) and mainstream smoke (smoke ex-haled by a person), resulting in the production of numerous gasses and particulate matter^1^. The International Agency for Research on Cancer stated that SHS exposure has a cancerogenic nature^2-4^. SHS exposure increases risk of developing a range of serious illnesses, including lung cancer, cardiovascular disease, respiratory disease, and asthma. Other significant health outcomes such as eye irritation, nasal inflammation, and low birthweight are caused by secondhand smoke exposure^1^. In the post-Soviet and former pro-Soviet block countries of Estonia, Hungary, Latvia, Lithuania, Moldova, Russia, Belarus, Ukraine, and Kazakhstan, it is estimated that 61.0% of non-smoking children and 66.0% of non-smoking men and women are exposed to SHS in public places. In 2004, it is estimated that 99300 deaths resulted from exposure to SHS in the region^5^. A national tobacco study estimated that 22.9% adults smoke^6^ and 27.6% of adults are exposed to SHS in public eating places (bars, restaurants, cafes) in Kazakhstan^7^.

Article 159 of the Kazakhstan Health Act of 2009, despite being obliged by Article 8 of the World Health Organization’s Framework Convention on Tobacco Control to ban smoking in public areas, allows for designated smoking areas (DSAs) where food is served, if the owner chooses^8^, despite evidence that designated smoking areas do not protect non-smokers from SHS exposure^9,10^. Thus, Almaty, the largest city of Kazakhstan, has public eating venues with a diverse range of smoking restrictions including: venues with smoking and non-smoking zones, smoking allowed throughout the venue, completely smoke-free venues, and smoke-free venues with DSAs.

The goal of this study was to assess and compare air quality in public food-serving venues of Almaty with different smoking regulations by measuring indoor PM_2.5_ levels. Particularly, we aimed at analyzing whether separation of smokers and non-smokers by rooms/zones or by special designated smoking areas could protect people from SHS. The objectives were to: 1) measure and compare PM_2.5_ levels between non-smoking zones in venues (that permit smoking in separate smoking zones) to 100% smoke-free food-serving venue, 2) measure and compare PM_2.5_ levels in DSAs that are located in food-serving venues with venues where smoking is allowed throughout, and 3) evaluate the association of PM_2.5_ levels with smoker density. To the best of our knowledge, there are no published studies in post-Soviet countries with transitional economies that evaluated levels of PM_2.5_ in venues with different smoke-free policies, increasing the importance of the study.

## METHODS

For the current study, we utilized the Air Quality Index (AQI) of the U.S. Environmental Protection Agency (EPA) for linking PM_2.5_ with air quality, based on health impacts^11^. PM_2.5_ levels were measured using the TSI SidePak AM510 Personal Aerosol Monitor – a validated tool for real-time measuring of PM_2.5_ levels every minute^12-16^. Although our results express 30 minutes exposure values, the EPA AQI provides a good reference standard.

The study of food-serving venue facilities did not require approval because it did not include human study participants and was not considered human subject research. Only facility internal structures, counts of burning tobacco, and environmental particulate matter measurements were recorded.

### Venue selection

The indoor air quality monitoring study was conducted from September to October 2017 in the city of Almaty, Kazakhstan. A total of 29 restaurants, cafes, and bars were selected from a list of popular food-serving venues in the densest areas in each of the 8 administrative regions of Almaty^17^ and coded by the numbers and first letters. Researchers previously assessed smoking restrictions and peak hours of operation by phone for each venue. Three to four venues from each region were selected for the study.

At least two venues from each district were selected. Among the 29 venues, 5 were 100% smoke-free, 15 had mixed smoking policies (smoking and non-smoking zones, or separate designated smoking areas) and 9 allowed smoking throughout the venue. The research team included a Campaign for Tobacco- Free Kids trainer and volunteers from the Kazakhstan Smoke-free coalition who selected the venues using criteria that included the venue’s smoking policy, peak hours of operation and average number of customers.

### Materials

The interior dimensions of the venues were measured using a Zircon DM 840 laser. Because some food-serving venues have different wall shapes, the volumes of venues were calculated based on laser measurements. The volume of some venues was more difficult to calculate due to their odd-shaped interior walls and ceilings and therefore were calculated separately using specialized diagrams. This approach has been used in similar studies^12,15,16,18^.

### Data collection

A total of 29 venues were observed during the research period. According to the type of venue smoking policies and room selection, rooms were divided into six types: 100% smoke free (SF), smoking throughout the venue (SV), non-smoking rooms with designated areas for smokers (NR), designated areas for smokers (DSA), venues that allow smoking zones (SZ), and non-smoking zones (NZ). The last category usually did not have a door or any separation between rooms for smokers and non-smokers.

A researcher from the Campaign for Tobacco-Free Kids operated the SidePak AM510 Personal Aerosol Monitor and measured the volume of the room by utilizing a laser. A trained person as data collector counted the number of smokers. Air for analysis was collected through a Tygon tubing that was connected to the AM510. Researchers did a thorough walk-through each venue. Food-serving venues are busiest on weekends at late evening hours, so the data collectors visited venues on Thursdays, Fridays, Saturdays and Sundays from 7 pm until midnight.

Before data collection inside the venue, PM_2.5_ exposure was evaluated for 5 min outside the building. Data were collected inside the venue for at least 30 min, and outside the venue for 5 min. If a venue had smoking and non-smoking rooms, data were collected for 30 min in each room. Between each room type, the recalibration was done during 5 min outside the building.

Data collection was incognito, no one was approached inside the venue. Researchers purchased food and drinks in the venue. The SidePak AM510 was carried in a small handbag, and inconspicuously was left on the shoulder or on a table near the researcher. For each venue the name of the venue, type of venue, time of entry and exit, the volume of the room, the number of people and counts of burning cigarettes and hookahs taken in 15-minute intervals, and other possible sources of PM_2.5_ such as lit candles or fumes from the kitchen were recorded.

### Data analysis

For the analysis, each room type in the venue was utilized as a separate unit of analysis. According to the division of food-serving venues by smoking policies and rooms, six room types were analyzed: SF (100% smoke free – where smoking is strictly prohibited), SV (smoking allowed throughout the venue), SZ (smoking zone inside the venue) and NZ (non-smoking zones inside the venue), NR (non-smoking room in venue with DSA), and DSA (designated areas for smokers).

The average smoker density was calculated by taking the mean number of burning tobacco products per 100 m^3^. Burning tobacco products included both cigarettes and hookahs. No electronic cigarettes were observed in the selected venues. The mean number of burning tobacco products (mean number of people smoking) inside a room was estimated by counting the number of burning tobacco products at the start of the observation period, and at 15 minutes and 30 minutes later. The sum of tobacco burning products per m^3^ was divided by three to calculate the mean. The measurements for SidePak AM510 were made by excluding the first and the last minute of the PM_2.5_ data for each venue, while adjusting for the calibration factor of 0.32^18-20^.

Analysis was conducted using the STATA SE/12.0 software package. Non-parametric tests were applied due to small sample size and lack of normality. The Kruskal-Wallis test was utilized to assess whether the mean PM_2.5_ concentration levels across room types differed. To determine differences for median PM_2.5_ concentrations, eight comparison groups for room types were analyzed. The Wilcoxon signed rank test was conducted for comparing rooms (NR and DSA; SZ and NZ) and Mann-Whitney U test for venues (SZ and DSA; NZ and NR; SF and NZ; SF and NR; SV and SZ; SV and DSA). The association between the mean smoker density and mean PM_2.5_ concentrations were explored using the Spearman correlation. All tests were conducted with an α value of 0.05.

## RESULTS

A total of 44 rooms within the selected 29 venues were included in the analysis. The results are presented in Table 1, for descriptive variables including: the volume of the venue, the mean smoker density, the mean PM_2.5_ concentration, the venue’s smoking policy, and the type of room.

**Table 1:** Descriptive statistics and PM_2.5_ concentrations for each venue by room smoking status

*Venue name*	*Type of room*	*Venue size (m^3^)*	*Mean number of people*	*Mean tobacco burning products*	*Mean PM_2.5_ (μg/m^3^)*	*Mean smoker density (mean burning products per 100 m^3^)*
**SF: Smoke-free (smoking is strictly prohibited)**						
1 CV	SF	76.8	2.0	0	13.8	0
2 St	SF	377.2	22.0	0	3.5	0
3 CT	SF	642.6	41.3	0	63.5	0
4 MS	SF	77.0	7.3	0	17.3	0
5 RBG	SF	476.0	18.3	0	33.0	0
Average		330.0	18.2	0	26.2	0
**NZ: Non-smoking zone of the venue (venue has smoking and non-smoking zones)**						
6 NB	NZ	72.9	6.6	0	51.1	0
7 Tu	NZ	495.0	12.0	0	91.1	0
8 RJ	NZ	126.0	13.0	0	70.9	0
9 MZ	NZ	54.0	13.3	0	75.4	0
10 Ve	NZ	115.2	11.0	0	32.8	0
11 Lo	NZ	312.0	16.3	0	51.2	0
12 Mo	NZ	544.5	9.3	0	22.7	0
Average		245.6	11.6	0	56.5	0
**SZ: Smoking zone of the venue (venue has smoking and non-smoking zones)**						
6 NB	SZ	186.2	11.3	0.6	258.5	0.4
7 Tu	SZ	742.5	19.0	2.6	315.1	0.4
8 RJ	SZ	79.8	4.0	1.6	133.6	2.1
9 MZ	SZ	102.5	13.0	2.6	285.2	2.6
10 Ve	SZ	103.3	7.0	0.3	55.9	0.3
11 Lo	SZ	124.2	5.3	2.6	183.9	2.2
12 Mo	SZ	544.5	29.0	1.3	42.0	0.2
Average		269.0	12.6	1.7	182.0	1.2
**SV: Smoking is allowed throughout the venue**						
13 LIH	SV	995.2	78.0	5.6	119.6	0.6
14 Ko	SV	155.1	16.0	5.3	581.8	3.4
15 Bo	SV	665.8	8.0	2.6	47.4	0.4
16 NS	SV	270.0	23.0	1.6	65.2	0.6
17 Zh	SV	360.0	33.3	4.6	178.3	1.3
18 ZK	SV	90.0	8.6	1.6	183.9	1.9
19 Do	SV	396.0	5.0	0.6	103.5	0.2
20 Te	SV	753.7	24.6	6.3	115.3	0.8
21 Ni	SV	551.0	24.6	3.0	227.5	0.5
Average		470.8	24.6	3.5	180.3	1.1
**NR: Non-smoking room of the venue with DSA**						
22 Ro	NR	434.8	9.3	0	67.5	0
23 Al	NR	1174.7	55.3	0	78.3	0
24 FV	NR	1926.4	123.0	0	144.4	0
25 Ba	NR	217.0	33.6	0	20.6	0
26 Gu	NR	195.0	19.3	0	33.2	0
27 Ko	NR	465.5	16.6	0	66.7	0
28 Mu	NR	296.6	16.3	0	54.3	0
29 Ch	NR	484.5	58.0	0	235.8	0
Average		649.0	41.4		87.6	
**DSA: Designated smoking area inside the venue**						
22 Ro	DSA	50.9	2.3	1.6	360.3	3.3
23 Al	DSA	36.7	5.3	5.3	782.5	14.5
24 FV	DSA	33.6	4.0	4.0	585.8	11.9
25 Ba	DSA	17.6	3.6	3.6	464.6	20.8
26 Gu	DSA	26.3	4.0	4.0	422.4	15.2
27 Ko	DSA	19.2	1.6	1.6	433.4	8.7
28 Mu	DSA	102.8	1.0	1.0	172.7	1.0
29 Ch	DSA	24.3	5.6	5.6	1961.6	23.3
Average		38.9	3.5	3.4	647.9	12.3

The mean customer occupancy of a room varied from 1 to 78 people. Non-smoking rooms (NR) that had separate areas for smoking (DSAs) were the most crowded, with a mean of 41.4 persons per room, followed by smoking venues with 24.6 persons per room and smoke-free venue with 18.2 persons per room. Non-smoking and smoking zones inside venues had almost the same occupancy with 12.6 and 11.6 persons per room, respectively. A mean of 3.5 persons was observed in DSAs, which had an average size of 38.9 m^3^. No tobacco product use was observed in smoke-free venues and non-smoking zones. Smoking venues and DSAs were observed to have a mean range of 1.7 to 3.5 burning cigarettes/m^3^. The mean density of burning tobacco products in smoking rooms ranged from 1.2 to 12.3 per 100 m^3^ inside DSAs. Most of the observed venues were larger than 250 m^3^.

Table 2 shows the mean smoker density and PM_2.5_ by room type and provides the corresponding U.S. EPA AQI rankings for air quality for particulate matter concentrations. The results show that 100% smoke-free venues had the lowest mean PM_2.5_ concentration of 26.2 μg/m^3^, which corresponds to moderate levels of air quality. In contrast, non-smoking zones in venues that allowed DSAs had a mean PM_2.5_ concentration of 56.5 μg/m^3^, corresponding to unhealthy air quality. Non-smoking rooms in venues that have DSAs as separate rooms also had unhealthy air quality, with mean PM_2.5_ concentrations of 87.6 μg/m^3^. Designated smoking areas had a mean PM_2.5_ concentration of 647.9 μg/m^3^, a hazardous level of air quality, while food-serving venues with either a smoking zone or which allowed smoking throughout the venue had mean PM_2.5_ concentrations of 180.3–182.0 μg/m^3^, ranking as a very unhealthy level of air quality. The mean PM_2.5_ concentration for the non-smoking zones (56.5 μg/m^3^) was 2.1 times higher than the 100% smoke-free venues (26.2 μg/m^3^). The mean PM_2.5_ concentration level of 647.9 μg/m^3^ inside DSAs was 3.6 times greater than the mean PM_2.5_ concentration in venues that allow smoking throughout the venue (180.3 μg/m^3^) and almost 25 times higher than in smoke-free settings (26.2 μg/m^3^).

**Table 2:** Summary statistics (mean±SD) for each venue/room category and corresponding U.S. EPA AQI rankings, by PM_2.5_ (μg/m^3^) [24-hour]

*Smoking status*	*N*	*Mean smoker density*	*Mean PM_2.5_ (μg/m^3^)*	*Median PM_2.5_ (μg/m^3^)*	*U.S. EPA AQI ranking*
100% Smoke-free venue (SF)	5	0	26.2±23.4	17.3	Moderate
Non-smoking zones (NZ) of the venues with smoking zones	7	0	56.5±24.2	51.2	Unhealthy
Non-smoking room of the venue with DSA (NR)	8	0	87.6±70.4	67.1	Unhealthy
Smoking throughout the venue (SV)	9	1.1±1.0	180.3±161.3	119.6	Very unhealthy
Smoking zones (SZ) of the venues with non-smoking zone	7	1.2±1.1	182.0±109.6	184.0	Very unhealthy
Designated smoking rooms in smoke-free venues (DSA)	8	12.3±7.9	647.9±559.0	449.0	Hazardous

The mean customer occupancy of a room varied from 1 to 78 people. Non-smoking rooms (NR) that had separate areas for smoking (DSAs) were the most crowded, with a mean of 41.4 persons per room, followed by smoking venues with 24.6 persons per room and smoke-free venue with 18.2 persons per room. Non-smoking and smoking zones inside venues had almost the same occupancy with 12.6 and 11.6 persons per room, respectively. A mean of 3.5 persons was observed in DSAs, which had an average size of 38.9 m^3^. No tobacco product use was observed in smoke-free venues and non-smoking zones. Smoking venues and DSAs were observed to have a mean range of 1.7 to 3.5 burning cigarettes/m^3^. The mean density of burning tobacco products in smoking rooms ranged from 1.2 to 12.3 per 100 m^3^ inside DSAs. Most of the observed venues were larger than 250 m^3^.

**Figure 1:**
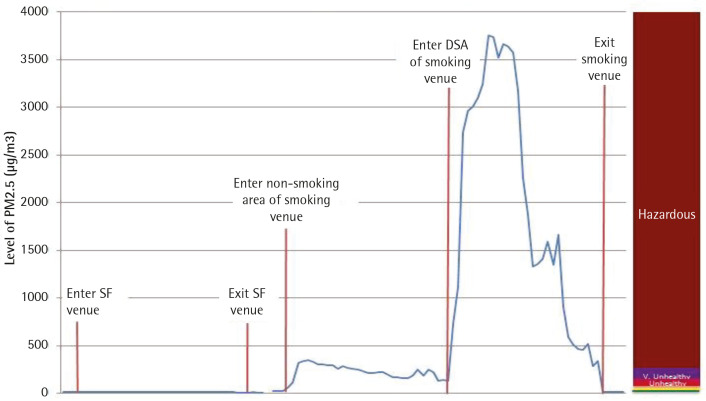
Minute-by-minute PM_2.5_ levels: 100% smoke-free food-serving venues vs smoking venues with non-smoking areas and designated smoking areas, Sept–Oct 2017

Figure 1 shows the minute-by-minute comparison of PM_2.5_ levels in 100% smoke-free food-serving venues compared to smoking venues with non-smoking areas and those with designated smoking areas.

The graph indicates that inside the smoke-free food-serving venues, PM_2.5_ levels were consistently low. The entrances to non-smoking zones of venues that also had smoking zones had higher levels of PM_2.5_, ranging from approximately 200 to 300 μg/m^3^, which indicates unhealthy air quality. The entrance to a selected DSA had an average PM_2.5_ level of 3600 μg/m^3^, which is 14.4 times greater than the average PM_2.5_ levels of the non-smoking areas of the venues.

The mean smoker density in venues where smoking was not allowed (SF, NZ, NR) was zero, unlike venues that provided smoking allowed zones and where smoking was allowed throughout the venue, which had similar values of 1.1 and 1.2 per 100 m^3^, respectively. DSAs had the highest mean smoker density of 12.3. Smoke-free venues (SF) have the lowest mean PM_2.5_ concentration of 26.2 μg/m^3^. The mean PM_2.5_ concentrations of non-smoking zones (NZ) ranged from 32.3 to 80.7 μg/m^3^, with an overall mean of 56.5 μg/m^3^. Non-smoking rooms of venues with DSAs (NR) had a mean PM_2.5_ concentration of 87.6 μg/m^3^, while DSAs in these venues had a mean of 647.9 μg/m^3^.

Restaurants where smoking is allowed throughout the venue (SV) or provide smoking zones (SZ) showed similar mean PM_2.5_ concentrations around 180.3 μg/m^3^. Across all room types, the mean PM_2.5_ concentrations ranged from the lowest value 26.2 μg/m^3^ in SFs to the highest value of 647.9 μg/m^3^ in DSAs. Based on the U.S. EPA AQI, only smoke-free venues had moderate levels of air pollution, while other room types showed unhealthy (NZ, NR), very unhealthy (SV, SZ) or hazardous levels (DSA) of air pollution. The results of the Spearman correlation analysis indicate a strong correlation between smoker density and PM_2.5_ concentrations across all room types (r=0.80; n=44; p<0.01).

A Kruskal-Wallis H test was conducted to discover whether median PM_2.5_ concentrations were different among room types SF(5), NZ(7), NR(8), SV(9), SZ (7), and DSA(8). The test showed that there was a statistically significant difference in median PM_2.5_ concentrations across all six room types (p=0.0001). The Wilcoxon signed-rank test within the venues showed that the median PM_2.5_ concentration in smoking zones (SZ) found no difference from non-smoking zones (NZ) (p=0.062). The same test showed that the median PM_2.5_ concentrations between non-smoking rooms and DSAs within the same venues are different (p=0.01). The Mann-Whitney U test showed a statistically significant difference between the median PM_2.5_ concentrations for smoking zones and DSAs (p=0.005). Median PM_2.5_ concentrations were not found to be different between smoking zones and venues that allow smoking throughout (p=0.56), between non-smoking zones and non-smoking rooms of the venues with DSAs (p=0.49), and between non-smoking zones and smoke-free venues (p=0.06). The Mann-Whitney U test also showed a difference in median PM_2.5_ concentrations between smoke-free venues and non-smoking rooms of the venues with DSAs (p=0.02) and with those venues that allow smoking throughout the venue and DSAs (p=0.01).

## DISCUSSION

To the best of our knowledge, this is the only study of the post-Soviet countries with transitional economies that provides PM_2.5_ concentrations categorized by food-serving venues with different smoke-free policies, from complete smoke-free environment to those venues with designated smoking areas. Results showed that limited smoke-free policies negatively affect air quality of indoor eating places in Almaty. According to Table 2, ‘moderately healthy’ air quality was observed in smoke-free venues, while all other rooms in food-serving venues showed poor air quality levels. Despite the lack of tobacco burning products, non-smoking zones and non-smoking rooms in venues with DSAs showed ‘unhealthy’ air quality levels based on the U.S. EPA AQI Rankings for PM_2.5_. Venues were smoking is allowed throughout and venues with smoking zones had air quality ranked as ‘very unhealthy’. The poorest air quality was measured in DSAs, corresponding to ‘hazardous’ levels. The highest density of people and the smallest volumes for rooms were observed for DSAs, where air quality was hazardous for human health.

According to our findings, the implementation of separate smoking zones or DSAs does not substantially improve air quality inside food-serving venues. The mean PM_2.5_ concentrations in the non-smoking zone and non-smoking room in venues with DSAs are unhealthy and are more than two times and three times higher than in smoke-free venues, respectively. Wilcoxon test helped to establish the difference in median PM_2.5_ concentrations between smoking zone and non-smoking zones of the venue. This indicates that food-serving venues that have separate zones for smokers and non-smokers do not substantially protect the non-smokers from secondhand smoke. Comparison of the median PM_2.5_ concentrations between non-smoking zones and non-smoking rooms in venues with DSAs showed no statistical differences. This also indicates that DSAs also do not substantially protect non-smokers from secondhand smoke. PM_2.5_ concentrations in DSAs are 3.5 times higher than in venues where smoking is allowed throughout the venue. These findings indicate that DSAs do not protect employees and non-smoking patrons from exposure to unhealthy air quality.

Results suggest that the substantial part of PM_2.5_ concentrations are due to cigarette and/or hookah smoking. Our findings determined that the higher the smoker density the poorer the air quality in the venue.

The results of the study are consistent with those from other publications that analyzed air quality levels of hospitality venues by measuring particulate matter under diverse smoking policies. The analysis of 95 hospitality venues in Switzerland, showed four times higher concentrations of PM_2.5_ in non-smoking rooms than in smoke-free environments, despite separation of smokers from non-smokers^21^. In Sydney, a study of 17 gaming clubs comparing PM_2.5_ concentrations in designated smoking areas with non-smoking areas found that non-smoking areas still had almost half of the particulate matter concentrations of the designated smoking areas. The study concluded that DSAs provide limited to no protection from secondhand smoke^10^.

### Limitations

Two limitations are present in the study. First, we had observed only 29 places of Almaty due to time constraints and financial restrictions. Although we had covered all possible variations of existing smoke-free policy, such research should be done in other big cities to be able to generalize results to the whole country. The second limitation is the presence of both cigarettes and hookahs at the observation sites. Thus, it is not clear which tobacco product contributed most to the air pollution inside the venues. However, this limitation is difficult to avoid due to the mixture of smoke as most venues propose both hookahs and cigarettes to patrons.

## CONCLUSIONS

Smoking policies for food-serving venues that allow designated smoking areas are ineffectual in protecting people from secondhand tobacco smoke exposure. Non-smoking rooms in venues with DSAs had two times higher air pollution levels than smoke-free venues. A smoke-free policy in venues is the only way to effectively protect non-smokers from secondhand smoke in hospitality settings. Non-smoking zones and venues that were compliant to the law and restricted smoking to DSAs only, had unhealthy air quality in non-smoking areas and hazardous levels of PM_2.5_ inside DSAs due to tobacco products. It proves the low efficiency of a smoking ban with exemptions. Complete smoking ban in food-serving venues should be in place to fully protect people from hazardous air quality conditions in Kazakhstan.
